# Distinct Dysfunctional States of Circulating Innate-Like T Cells in Metabolic Disease

**DOI:** 10.3389/fimmu.2020.00448

**Published:** 2020-03-13

**Authors:** Yanyan Li, Katherine Woods, Amber Parry-Strong, Regan J. Anderson, Celina Capistrano, Aurelie Gestin, Gavin F. Painter, Ian F. Hermans, Jeremy Krebs, Olivier Gasser

**Affiliations:** ^1^Malaghan Institute of Medical Research, Wellington, New Zealand; ^2^School of Medicine, University of Otago, Wellington, New Zealand; ^3^Ferrier Institute, Victoria University of Wellington, Wellington, New Zealand; ^4^High-Value Nutrition National Science Challenge, Auckland, New Zealand

**Keywords:** MAIT cells, iNKT cells, Vδ2^+^ T cells, obesity, type 2 diabetes

## Abstract

The immune system plays a significant role in controlling systemic metabolism. Innate-like T (ILT) cells in particular, such as mucosal-associated invariant T (MAIT) cells, invariant natural killer T (iNKT) cells and γδ T cell receptor expressing cells, have been reported to promote metabolic homeostasis. However, these different ILT cell subsets have, to date, been generally studied in isolation. Here we conducted a pilot study assessing the phenotype and function of circulating MAIT, iNKT, and Vδ2^+^ T cells in a small cohort of 10 people with obesity and type 2 diabetes (T2D), 10 people with obesity but no diabetes, and 12 healthy individuals. We conducted phenotypic analysis by flow cytometry *ex vivo*, and then functional analysis after *in vitro* stimulation using either PMA/ionomycin or synthetic agonists, or precursors thereof, for each of the cell-types; use of the latter may provide important knowledge for the development of novel therapeutics aimed at activating human ILT cells. The results of our pilot study, conducted on circulating cells, show clear dysfunction of all three ILT cell subsets in obese and obese T2D patients, as compared to healthy controls. Importantly, while both iNKT and Vδ2^+^ T cell dysfunctions were characterized by diminished IL-2 and interferon-γ production, the distinct dysfunctional state of MAIT cells was instead defined by skewed subset composition, heightened sensitivity to T cell receptor engagement and unchanged production of all measured cytokines.

## Introduction

Unconventional, innate-like T (ILT) cells are generally defined as subsets of T lymphocytes recognizing conserved, non-peptide antigens in the context of monomorphic major histocompatibility complex (MHC) related or unrelated molecules. In humans, these include mucosal-associated invariant T (MAIT) cells, invariant natural killer T (iNKT) cells and Vγ9^+^Vδ2^+^ T cells, which feature prominent roles in microbial immunity and characteristic PLZF (promyelocytic leukemia zinc finger, Zbtb16)-dependent innate-like effector programs (1, 2). Specifically, MAIT cells target riboflavin metabolites derived from bacteria or yeast (3) presented in the context of MHC-related molecule 1 (MR1) (4, 5), while iNKT cells and Vγ9^+^Vδ2^+^ T cells recognize microbial lipid antigens presented by CD1d (6), and bacterial and neoplastic phosphoantigens (7, 8) bound to butyrophilin-3A1 (9), respectively. Interestingly, ILT cells not only patrol mucosal sites to prevent microbial infection but appear to home to adipose tissue (AT) as well. Current experimental and clinical evidence suggests that ILT cells promote immune homeostasis and thermogenesis in lean AT, but may initiate or exacerbate AT inflammation in the context of obesity and type 2 diabetes (10–18). Overall, the relative invariance of ILT cell specificity across the human population and their general involvement in AT homeostasis may provide novel targets or approaches to therapy, as exemplified by the significant weight loss and successful restoration of glycemic control observed in obese mice after administration of the classical iNKT cell agonist α-galactosylceramide (α-GC) (10, 15, 19). However, ILT cells appear in different frequencies in mice as compared to humans. MAIT cells commonly outnumber iNKT cells by an order of magnitude in humans, while the opposite is true in mice. Furthermore, the dominant subset of PLZF^+^ γδ T cells express Vγ9 and Vδ2 in humans, but these cells have no equivalent in mice with respect to antigen-specificity, although there are thermogenic AT-resident PLZF^+^ γδ T cells in mice that express Vδ6 (16). Few studies have investigated Vγ9^+^Vδ2^+^ T cells in the context of metabolic disease in humans (16, 20).

To further our understanding of ILT cell biology in human metabolic disease and start the development of ILT cell-based therapeutics, as currently employed for cancer immunotherapy by us and others (21, 22), we conducted a pilot study aimed at providing the first side-by-side comparison of metabolic disease-induced functional changes to circulating MAIT, iNKT, and Vδ2^+^ T cells [predominantly paired with Vγ9 in humans (23)]. We assess ILT cells within PBMC samples in this study, since previous studies have demonstrated ILT cell phenotypes in PBMC to be comparable with AT (14). As important first step toward therapeutic application, we also provide a differentiating analysis of MAIT, iNKT, and Vδ2^+^ T cell cytokine secretion upon general activation (i.e., combination of phorbol myristate acetate (PMA) and ionomycin) as well as stimulation using the MAIT agonist precursor 5-amino-6-D-ribitylaminouracil [5-A-RU (24)], and the iNKT and Vγ9^+^Vδ2^+^ T cell agonists α-galactosylceramide [α-GC; used clinically by us and others (21, 25)] and 4-bromo-3-hydroxy-3-methylbutyl diphosphate [BrHPP; the only clinically tested phosphoantigen (26, 27)], respectively.

## Methods

### Patients and Healthy Control Subjects

This study included 32 participants divided into 3 groups; overweight participants with type 2 diabetes (T2D) (*n* = 10, 5 male/5 female, aged 64.4 ± 2.8 years) with body mass index (BMI) = 34.0 kg/m^2^ ± 1.5; overweight participants with normal glucose tolerance (*n* = 10 5 male/5 female, aged 45.6 ± 3.1 years) with BMI = 37.8 ± 1.8; and healthy control participants (*n* = 12, 6 male/6 female, aged 49.3 ± 4.5 years). All participants with T2D were taking metformin and 80% (8 out of 10) were also taking insulin. Blood samples of participants and healthy controls were either collected at the Center for Diabetes, Endocrine and Obesity Research, Wellington Regional Hospital or at the Malaghan Institute of Medical Research, Wellington New Zealand, after obtaining informed written consent. The study was approved by the New Zealand Health and Disability Ethics Committee (ref: 16/NTB/138) and conducted in adherence to standard biosecurity and institutional safety procedures.

### Isolation and *in vitro* Stimulation of PBMC

PBMCs were isolated from blood by means of density gradient centrifugation using Leucosep tubes (Sigma, St. Louis, MO). PBMCs were resuspended in 10% DMSO in heat-inactivated bovine serum (FBS; ThermoFisher Scientific, Rockford, IL) and stored in liquid nitrogen until use.

For non-specific stimulation, PBMCs were resuspended in an IMDM medium (ThermoFisher Scientific, Rockford, IL), supplemented with 5% heat-inactivated AB normal human serum (Sigma, St. Louis, MO), and plated in a 96-well round-bottom plate in a concentration ranging from 5 × 10^5^ to 2 × 10^6^ cell/mL. Cells were treated with phorbol myristate acetate (PMA, 50 ng/mL; Sigma, St. Louis, MO) and ionomycin (1 μg/mL; Sigma, St. Louis, MO) for 1 h at 37°C followed by addition of Brefeldin A (BFA, 10 μg/mL; Sigma, St. Louis, MO, USA) and Monensin (0.3 μg/mL; Sigma, St. Louis, MO) or left untreated for 6 h at 37°C. For antigen-specific stimulation, PBMCs were resuspended and plated as described above and incubated in the presence or absence of 5-A-RU (28) (10 μM), α-GC (29) (100 ng/mL) or BrHPP (5 μM; kindly provided by Innate Pharma, Marseille, France) for 2 h before the addition of BFA (10 μg/mL) and monensin (0.3 μg/mL). Although 5-OP-RU is the cognate antigen for MAIT cells, it is highly unstable, and 5-A-RU was administered as a precursor, relying on endogenous methyl glyoxal to generate 5-OP-RU (28).

The PBMC cultures were incubated at 37°C overnight before flow cytometric analysis.

### Flow Cytometry

Samples were incubated with Zombie Aqua™ dye (BioLegend, San Diego, CA) for 15 min in the dark at room temperature, to distinguish live cells from dead, and were subsequently washed with FACs buffer. Privigen normal immunoglobulin (CSL Behring, Bern, Switzerland) was used as an Fc-block in staining and wash buffers. Antibody staining was carried out for 20 min in the dark on ice. Fixation and permeabilization was performed using the Cytofix/Cytoperm kit (BD, San Jose CA), as instructed by the manufacturer. Intracellular staining was for 30 min at room temperature protected from light. Antibodies were sourced from BioLegend unless otherwise mentioned. The following antibodies were used throughout: anti-CD19 (HIB19), anti-CD3 (UCHT1), anti-CD4 (OKT4), anti-CD8 (RPA-T8, BD), anti-CD161 (HP-3G10), anti-TCR Vα7.2 (3C10), anti-TCR Vα24-Jα18 (6B11), anti-TCR Vδ2 (B6), anti-CD25 (M-A251), anti-CD69 (FN50), anti-IL-2 (MQ1-17H12), anti-IL-17 (BL168), anti-IL-4 (MP4-25D2), anti-IFN-γ (4S.B3), anti-Granzyme B (GB11, BD).

Stained cells were measured using an LSR-II flow cytometer (BD, Heidelberg, Germany). Data was analyzed with FlowJo 10 for Mac (Tree Star, Ashland, Ore).

### Statistics

All graphs and statistical tests were carried out using GraphPad Prism 8 (GraphPad, La Jolla, CA) or R software (Version 1.1.463). Statistical significance was assessed using one-way ANOVA with Tukey post-tests, Wilcoxon matched-pairs signed rank tests or non-parametric Mann-Whitney *U*-tests. Principle component analyses (PCA) were performed using prcomp function in R Studio and visualization of PCA was generated with ggbiplot R package.

## Results

### Metabolic Disease-Associated Differences in *ex vivo* Activation State and Subset Composition of ILT Cells

**Figure 1:**
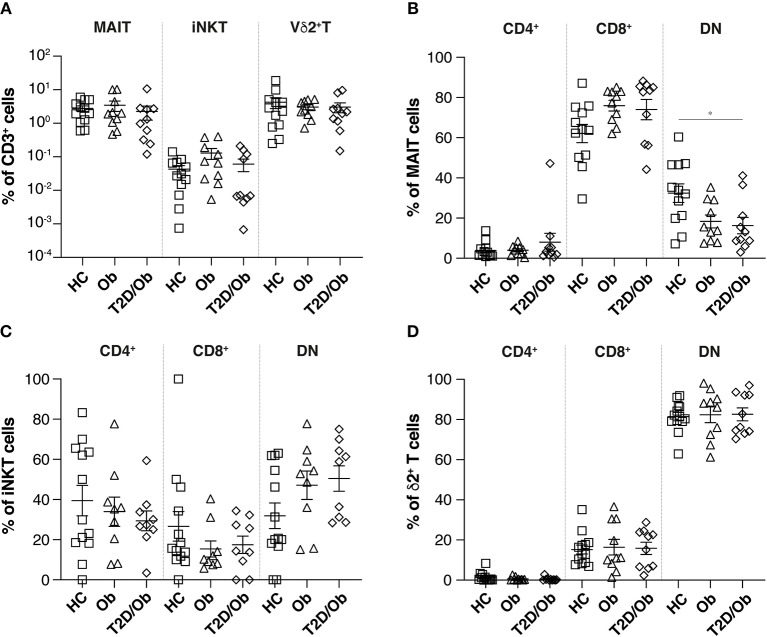
Frequency of innate-like T cells and associated subsets in obesity and type 2 diabetes. **(A)** Frequencies, as percentage of total CD3^+^ cells, of circulating MAIT, iNKT and Vδ2^+^ T cells in PBMC from healthy controls (HC), obese (Ob) and obese type 2 diabetic (T2D/Ob) patients. MAIT, iNKT and Vδ2^+^ T cells were identified as live CD19^−^CD3^+^TCRVα7.2^+^CD161^+^, CD19^−^CD3^+^TCRVα24-Jα18^+^ and CD19^−^CD3^+^TCRVδ2^+^ cells, respectively. **(B–D)** Frequencies of CD4^+^, CD8^+^, and CD4^−^CD8^−^ double negative (DN) innate-like T cell subsets, as percentage of total MAIT **(B)** iNKT **(C)** and Vδ2^+^ T cells **(D)**. Each symbol represents an individual. Plots show mean ± SEM. Statistics were calculated using one-way ANOVA with Tukey post-tests. *p < 0.05.

Under steady-state conditions, we observed no differences in the overall percentage of circulating MAIT, iNKT, or Vδ2^+^ T cells, between healthy controls (*n* = 12) and obese (*n* = 10) or obese T2D (*n* = 10) study participants (Figure 1A).

We assessed the composition of MAIT cell sub-populations, on the basis of three previously described subsets, namely: CD4^+^, CD8^+^, or CD4^−^CD8^−^ (double negative, DN) (30). Interestingly, we observed significantly higher percentages of DN MAIT cells in healthy controls, compared to patients with type 2 diabetes (T2D) (Figure 1B). This larger MAIT cell subset trended toward (*p* = 0.06) higher expression of the activation marker CD69 (Supplementary Figure 1), which might suggest that these cells represent recently or constitutively activated MAIT cells which have downregulated the surface expression of the CD8 co-receptor, as recently described (31). This hypothesis is further supported by the fact that after overnight *in vitro* culture, the percentage of CD69^+^ DN MAIT cells significantly dropped, with concomitant increase of the CD8^+^ fraction of CD69-expressing MAIT cells (Supplementary Figure 1). Of note, one person with diabetes had a significant proportion of CD4^+^ MAIT cells (47.2%). Such a high frequency of circulating CD4^+^ MAIT cells is not uncommon and has been reported before in healthy individuals (32). No significant differences in iNKT or Vδ2^+^ T cell subsets were observed (Figures 1C,D).

### Stimulus-Dependent Enhancement of CD25 and CD69 Expression on MAIT Cells From Obese and T2D Patients

**Figure 2:**
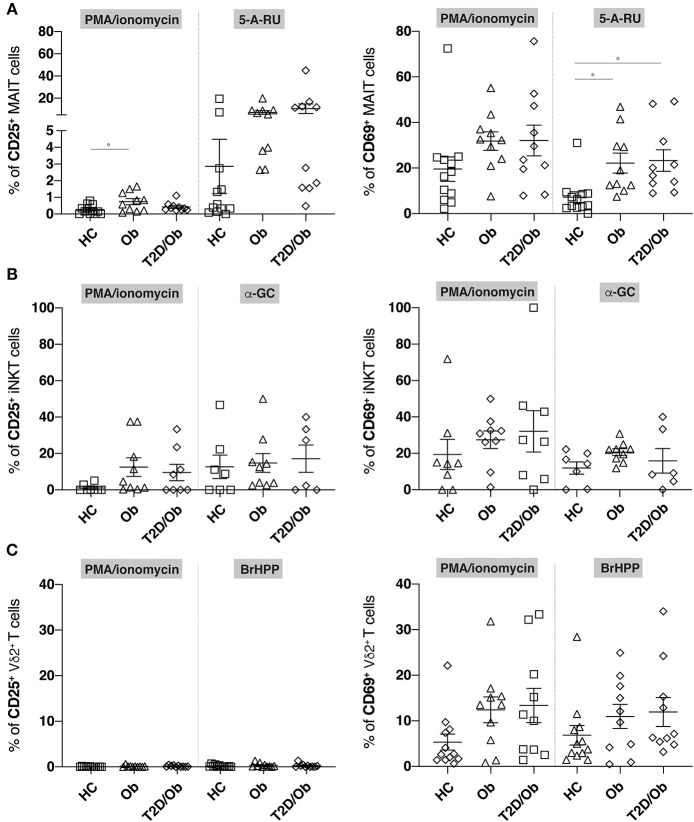
Frequency of CD25^+^ and CD69^+^ innate-like T cells in obesity and type 2 diabetes upon unspecific and antigen-specific activation. PBMC from healthy controls (HC), obese (Ob) and obese type 2 diabetic (T2D/Ob) patients were stimulated either non-specifically for 6 h with PMA/ionomycin or specifically for 20 h with the cognate antigens, or precursor thereof, for either MAIT cells (5-A-RU), iNKT cells (α-GC), or Vγ9^+^Vδ2^+^ T cells (BrHPP). Frequency of CD25^+^ (left panels) and CD69^+^ (right panels) cells are shown for **(A)** MAIT cells, **(B)** iNKT cells, and **(C)** Vδ2^+^ T cells. Each symbol represents an individual. Plots show mean ± SEM. Statistics were calculated using one-way ANOVA with Tukey post-tests. *p < 0.05.

We next compared the ability of MAIT, iNKT and Vδ2^+^ T cells from healthy controls, obese and obese/T2D patients to upregulate the activation markers CD25 and CD69 upon stimulation with PMA/ionomycin, or their respective stimulatory antigens. These were the riboflavin metabolite 5-A-RU for MAIT cells (4, 5), the lipid antigen α-GC for iNKT cells (15), and the phosphoantigen BrHPP for Vγ9^+^Vδ2^+^ T cells (7, 8). While the percentages of CD25- or CD69-expressing iNKT or Vδ2^+^ T cells did not differ between cohorts upon stimulation with either stimulus (i.e., PMA/ionomycin and α-GC or BrHPP, respectively), we observed an enhanced expression of CD69 on the surface of 5-A-RU-stimulated MAIT cells from obese and obese/T2D patients, compared to controls (Figures 2A–C). A similar trend was visible for CD25, which did not reach statistical significance. PMA/ionomycin-induced stimulation was unable to provide similar resolution regarding CD69 but induced higher CD25-expression in obese as compared to healthy individuals (Figure 2A).

### Distinct and Stimulus-Dependent Dysfunctional States of ILT Cells From Obese and T2D Patients

We next sought to determine how ILT function differs between healthy donors and patients with metabolic disease following activation of each cell subset. PBMC were again activated with either PMA/ionomycin, or the respective ILT-specific agonists, or precursor thereof, and cytokine/granzyme production assessed by intracellular staining.

**Figure 3:**
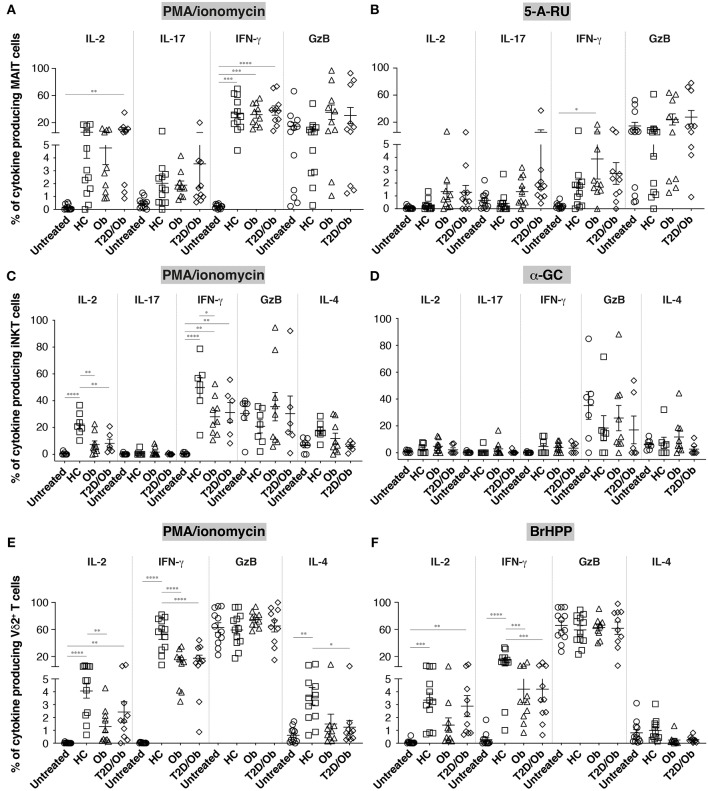
Frequency of cytokine-producing innate-like T cells in obesity and type 2 diabetes upon unspecific and antigen-specific activation. PBMC from healthy controls (HC), obese (Ob) and obese type 2 diabetic (T2D/Ob) patients were stimulated either non-specifically for 6 h with PMA/ionomycin **(A,C,E)** or specifically for 20 h with the individual cognate antigen, or precursor thereof, for either MAIT cells (5-A-RU; **B**), iNKT cells (α-GC; **D**), or Vγ9^+^Vδ2^+^ T cells (BrHPP; **F**). Untreated controls were incubated for 6 and 20 h, respectively. Each symbol represents an individual. Plots show mean ± SEM. Statistics were calculated using one-way ANOVA with Tukey post-tests. *p < 0.05, **p < 0.01, ***p < 0.001, ****p < 0.0001.

MAIT cell functionality did not differ significantly between groups, irrespective of stimulus used (Figures 3A,B and Supplementary Figure 3). The apparent increase in IL-17 production upon stimulation with 5-A-RU, previously reported using PMA/ionomycin (14), did not reach statistical significance.

We observed instead a remarkably similar impairment of iNKT and Vδ2^+^ T cells to produce IL-2 and IFN-γ in obese and/or obese/T2D patients (Figures 3C–F and Supplementary Figure 4). Interestingly, this impairment was only detectable in iNKT cells upon PMA/ionomycin stimulation, but showed in Vδ2^+^ T cells after both PMA/ionomycin and BrHPP stimulation. Vδ2^+^ T cells from obese/T2D patients also released significantly less IL-4 upon PMA/ionomycin stimulation, and to a lesser extent after BrHPP stimulation. Diminished IL-4 production in the context of obesity/T2D was also observable in iNKT cells, without reaching statistical significance. Principal component analyses further highlighted the IL-2/IFN-γ(/IL-4)-weighted separation of iNKT and Vδ2^+^ T cells from healthy controls vs. metabolic patients (restricted to PMA/ionomycin stimulation for iNKT cells; Supplementary Figure 2). Insulin therapy did not have any detectable impact on the ILT cell phenotypes (patients D4 and D6 did not receive insulin).

## Discussion

Overall, our pilot study partly supports previously described patterns of MAIT cell dysfunction in metabolic disease, with some discrepancies, but more importantly provides novel and comparative data related to obesity/T2D related dysfunction of human iNKT and Vδ2^+^ T cells, to date scarcely characterized.

Our study was apparently underpowered to recapitulate the previously described decrease in frequency, higher expression of activation markers and Th17 phenotype of peripheral MAIT cells in metabolic disease patients (12, 14). However, our results suggest that the altered frequency and activation state may be restricted to a particular MAIT cell subset, namely DN MAIT cells, which supports the proposed link between decreased peripheral MAIT cell frequency and activation-induced cell death (14) as DN MAIT cells have a characteristic pro-apoptotic gene signature (31). In this context, a notable discrepancy between our data and the study by Magalhaes et al. (14) is that we did not find MAIT cells from obese and obese/T2D patients to be refractory to T cell receptor (TCR) stimulation. On the contrary, we observed that 5-A-RU induced significant over-expression of CD69, and to a lesser extent CD25, in MAIT cells from patients as compared to controls. This difference may be explained by the choice of TCR stimulus, in our case synthetic 5-A-RU, the precursor of the MAIT agonist 5-(2-oxopropylideneamino)-6-D-ribitylaminouracil (5-OP-RU), while Magalhaes et al. used filtered bacterial supernatant. While in both cases antigen presenting cells are likely to mediate MR1-dependent presentation and MAIT cell activation, it is conceivable that MAIT cell agonists within bacterial supernatants are complexed to other solutes and may require active phagocytosis for MR1 loading and presentation, which is impaired in PBMC from metabolic disease patients (33), and thereby confound the results. Of note, the dose of 5-A-RU we used was sub-maximal, in that it did not drive significant CD69 upregulation by MAIT cells from healthy controls. Our results therefore suggest that MAIT cells from metabolic patients display heightened sensitivity to sub-maximal TCR engagement, an observation restricted to the activation regimen used herein and which may be easily masked by the presence of innate pattern recognition ligands found in bacterial supernatant. Interestingly, while cytokine production levels were in general lower in 5-A-RU- compared to PMA/ionomycin-stimulated MAIT cells, sub-maximal TCR stimulation via 5-A-RU induced similar levels of IL-17 to PMA/ionomycin stimulation in obese and obese/T2D patients. Although our study was not sufficiently powered to substantiate the previously reported metabolic disease-associated Th17 profile of MAIT cells, 5-A-RU could offer a technical advantage, in future studies, due to the fact that it induces significantly lower “background” IL-17-production in MAIT cells from healthy controls as compared to PMA/ionomycin treatment (*p* = 0.001, Wilcoxon matched-pairs signed rank test; data from Figures 3A,B).

Invariant NKT cells are arguably the most studied ILT subset in murine models of metabolic disease (10, 11, 13, 15, 17–19, 34–36). Our study complements existing knowledge by showing that circulating iNKT cells from obese and obese/T2D patients are impaired in their ability to secrete IL-2, IFN-γ, and to a lesser extent IL-4. This dysfunctional phenotype was remarkably mirrored by Vδ2^+^ T cells, which have to our knowledge never been functionally characterized in this context. IL-2 and IL-4 have been shown to mediate important regulatory and anti-inflammatory functions of iNKT cells in murine adipose tissue (10, 19, 36) and their reduced production observed here for both iNKT and Vδ2^+^ T cells, if reflecting their functional impairment *in situ* (i.e., adipose tissue), is likely to be mechanistically involved in metabolic derangement in humans as well. Future work will address the main limitation of our pilot study and provide the necessary comparative analyses in blood vs. adipose tissue. However, it is tempting to speculate that both iNKT and Vδ2^+^ T cells constitute potential therapeutic targets in human metabolic disease. Of particular relevance, a recent study has documented improved fasting blood glucose and insulin sensitivity in osteopenic women after alendronate treatment (37). Alendronate, like all bisphosphonates, inhibits osteoclastic bone resorption but also activates Vγ9^+^Vδ2^+^ T cells via the inhibition of the mevalonate pathway (38). Also, and perhaps related, obesity-related dysfunction of Vδ2^+^ T cells can be reversed *in vitro* by stimulation with 1-Hydroxy-2-methyl-buten-4yl 4-diphosphate (HDMAPP), a phosphoantigen analog of BrHPP (20).

Further investigation is needed to determine the precise role of MAIT cells in metabolic disease and discuss accordingly any potential therapeutic benefit of MAIT cell agonists in this setting. Indeed, IL-17 seems to have pleiotropic and opposing effects in adipose tissue (14, 16) and so might have MAIT cells. This future work may most conveniently be started by the administration of 5-A-RU, 5-OP-RU, or associated compounds in existing murine models of disease, including obesity/T2D as well as thermogenic adaption. The dearth of knowledge regarding interspecies overlap of ILT cell function and the consequence of differing frequencies of MAIT and other ILT cells in mice and human remain however a major potential pitfall in our efforts to explore the therapeutic targeting of ILT cells in metabolic disease.

## Data Availability Statement

All datasets generated for this study are included in the article/Supplementary Material.

## Ethics Statement

The studies involving human participants were reviewed and approved by New Zealand Health and Disability Ethics Committee (ref: 16/NTB/138). The patients/participants provided their written informed consent to participate in this study.

## Author Contributions

YL performed experiments, analyzed the data, and designed figures. KW reviewed the data and wrote the manuscript. AP-S, CC, and AG helped with experiments, experimental design or patient recruitment. RA and GP synthesized key reagents. IH and JK initiated the study. OG provided experimental design, reviewed the data and wrote the manuscript.

### Conflict of Interest

The authors declare that the research was conducted in the absence of any commercial or financial relationships that could be construed as a potential conflict of interest.

## Supplementary Material

Supplementary Figure 1 CD69 expression by MAIT cell subsets over time during *in vitro* culture. The expression of CD69 on unstimulated **(A)** CD8^+^, **(B)** CD4^+^, or **(C)** CD4^−^CD8^−^ double negative (DN) MAIT cell subsets was determined by flow cytometry following either 6 or 20 h (h) *in vitro* culture. Each pair of symbols and associated line represent an individual. Statistics were calculated using Wilcoxon matched-pairs signed rank tests (or Mann-Whitney *U*-test for the comparison between CD69^+^ DN MAIT cells from healthy controls and diabetic patients). ^**^*p* < 0.01, ^***^*p* < 0.001.

Supplementary Figure 2 Principal component analysis of cytokine secretion following ILT cell stimulation. **(A)** MAIT cells, **(B)** iNKT cells, or **(C)** Vδ2^+^ T cells were stimulated either non-specifically for 6 h, with PMA/ionomycin (left panels), or overnight with their individual cognate antigens or precursor thereof (5-ARU, α-GC, or BrHPP respectively; right panels). The percentage of each cell subset expressing IL-2, IL-4, IL-17, IFN-γ, or granzyme B in healthy controls (HC; red), obese (Ob; green), or type 2 diabetic/obese patients (T2D/Ob; blue) was determined by flow cytometry, and compared between the three groups by principal component analysis. D4 and D6 were the two T2D patients who did not receive insulin therapy.

Supplementary Figure 3 Representative gating strategy and flow cytometry plots of activated MAIT cells. **(A)** Overall gating strategy used to identify ILT cells and subsets thereof. MAIT cells activated with either **(B)** 5-A-RU or **(C)** PMA/iomomycin were identified as live CD19^−^CD3^+^TCR Vα7.2^+^CD161^+^ cells and cytokine production quantified by intracellular cytokine staining. Numbers indicate % of gated subsets.

Supplementary Figure 4 Representative flow cytometry plots of activated iNKT and Vδ2^+^ T cells. **(A)** iNKT cells, identified as live CD19^−^CD3^+^TCRVα24-Jα18^+^ cells, were activated with either PMA/ionomycin (upper row) or α-GC (lower row) and cytokine production quantified by intracellular cytokine staining. **(B)** Vδ2^+^ T cells, identified as live CD19^−^CD3^+^TCRVδ2^+^ cells, were activated with either PMA/ionomycin (upper row) or BrHPP (lower row) and cytokine production quantified by intracellular cytokine staining. Numbers indicate % of gated subsets.

## Abbreviations

γδ T cell: gamma-delta T cell
ILT cell: innate-like T cell
IFN-γ: interferon gamma
iNKT: invariant natural killer T
MAIT: mucosal-associated invariant T
MR1: major histocompatibility complex-related protein 1
T2D: type 2 diabetes.
