# Supplementary figures and images for: Distinct Dysfunctional States of Circulating Innate-Like T Cells in Metabolic Disease
Source: Front Immunol. 2020 Mar 13;11:448. doi: 10.3389/fimmu.2020.00448 (PMC7082397; doi:10.3389/fimmu.2020.00448)

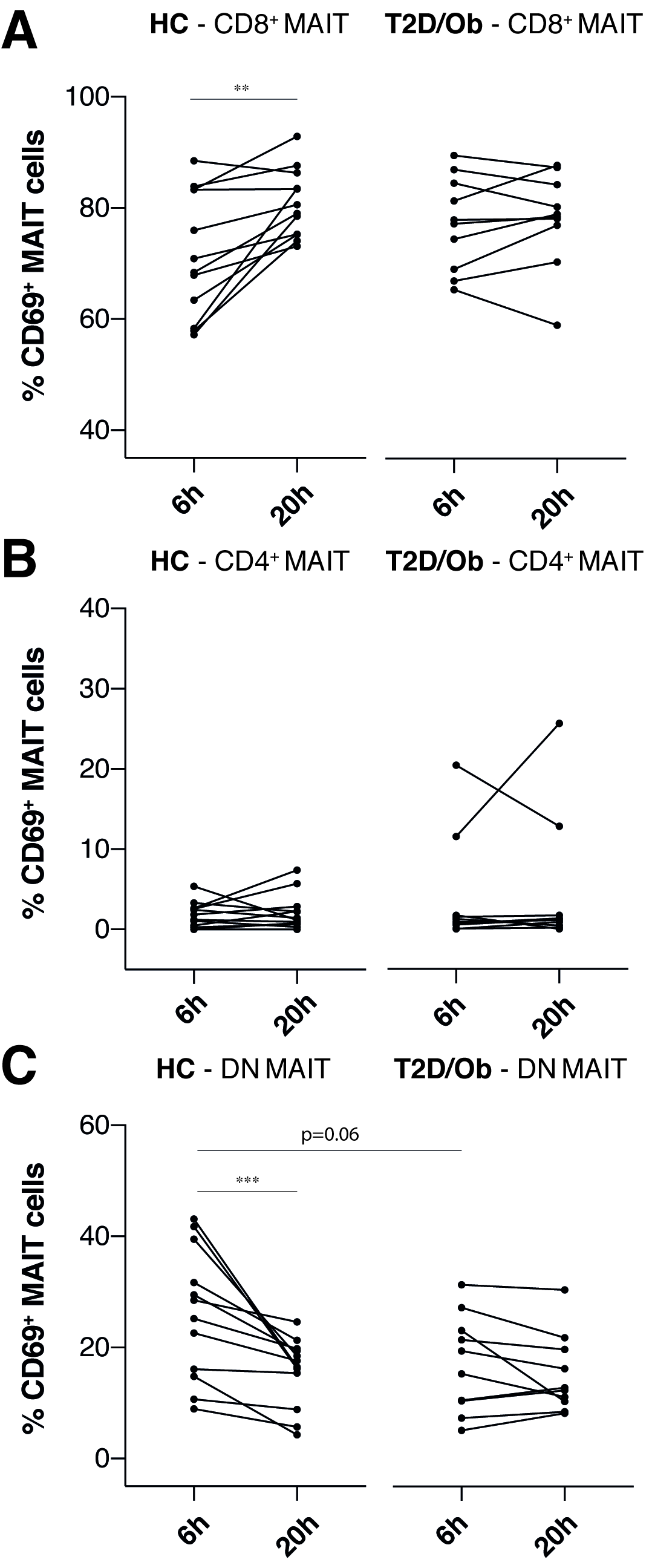

Supplement: Supplementary Figure 1 — CD69 expression by MAIT cell subsets over time during in vitro culture. The expression of CD69 on unstimulated (A) CD8+, (B) CD4+, or (C) CD4−CD8− double negative (DN) MAIT cell subsets was determined by flow cytometry following either 6 or 20 h (h) in vitro culture. Each pair of symbols and associated line represent an individual. Statistics were calculated using Wilcoxon matched-pairs signed rank tests (or Mann-Whitney U-test for the comparison between CD69+ DN MAIT cells from healthy controls and diabetic patients). **p < 0.01, ***p < 0.001. [file Image_1.TIF]

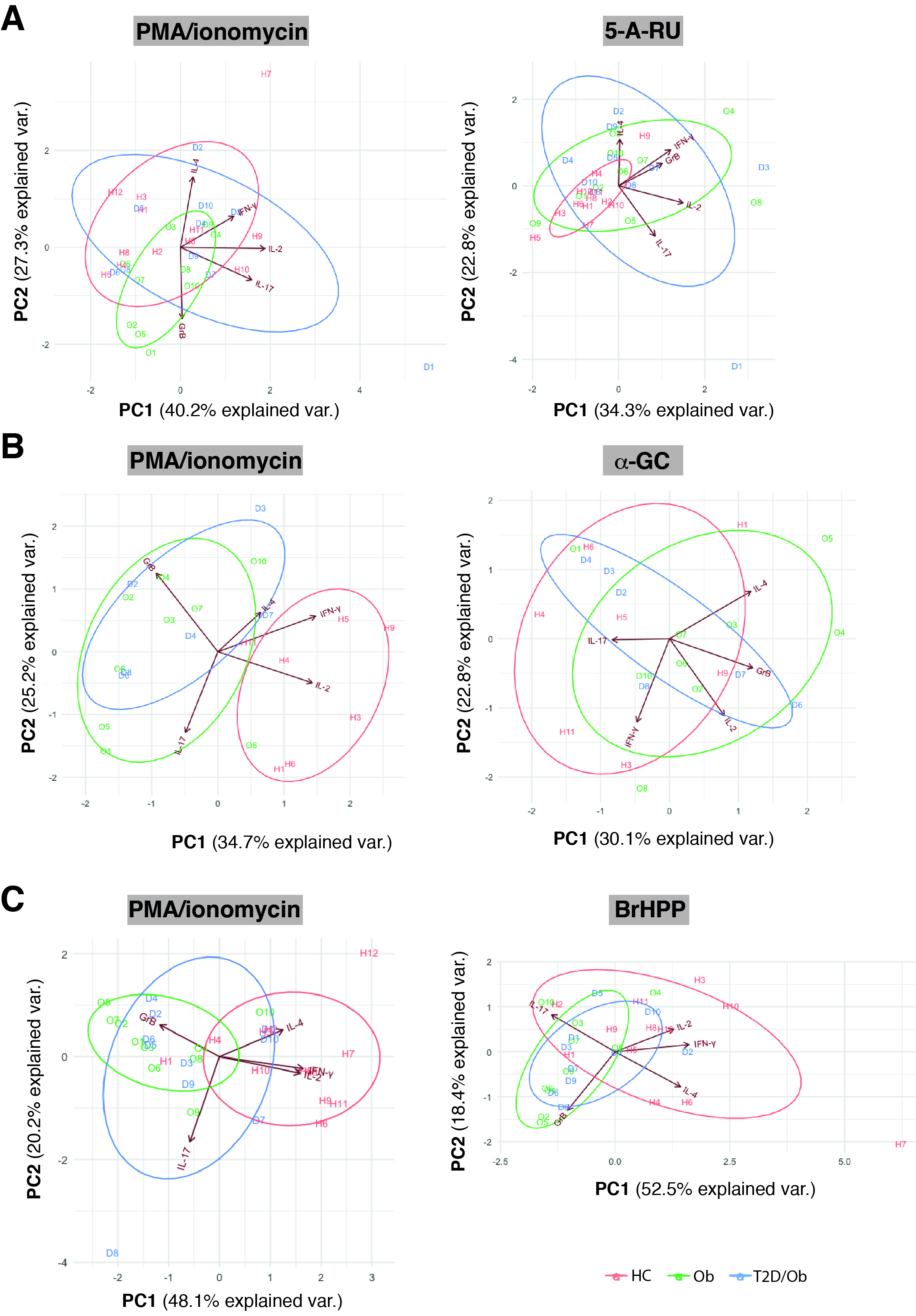

Supplement: Supplementary Figure 2 — Principal component analysis of cytokine secretion following ILT cell stimulation. (A) MAIT cells, (B) iNKT cells, or (C) Vδ2+ T cells were stimulated either non-specifically for 6 h, with PMA/ionomycin (left panels), or overnight with their individual cognate antigens or precursor thereof (5-ARU, α-GC, or BrHPP respectively; right panels). The percentage of each cell subset expressing IL-2, IL-4, IL-17, IFN-γ, or granzyme B in healthy controls (HC; red), obese (Ob; green), or type 2 diabetic/obese patients (T2D/Ob; blue) was determined by flow cytometry, and compared between the three groups by principal component analysis. D4 and D6 were the two T2D patients who did not receive insulin therapy. [file Image_2.JPEG]

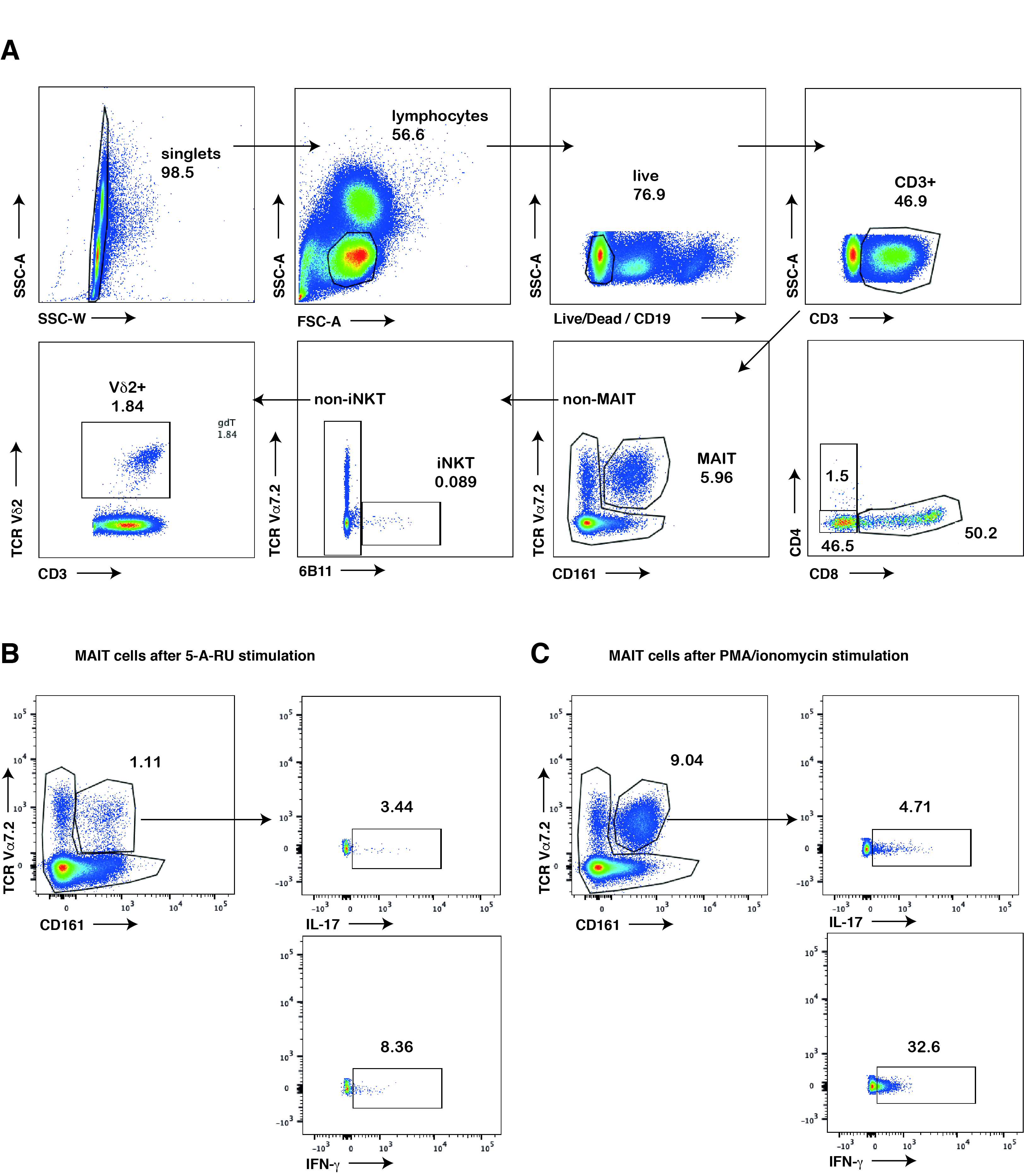

Supplement: Supplementary Figure 3 — Representative gating strategy and flow cytometry plots of activated MAIT cells. (A) Overall gating strategy used to identify ILT cells and subsets thereof. MAIT cells activated with either (B) 5-A-RU or (C) PMA/iomomycin were identified as live CD19−CD3+TCR Vα7.2+CD161+ cells and cytokine production quantified by intracellular cytokine staining. Numbers indicate % of gated subsets. [file Image_3.JPEG]

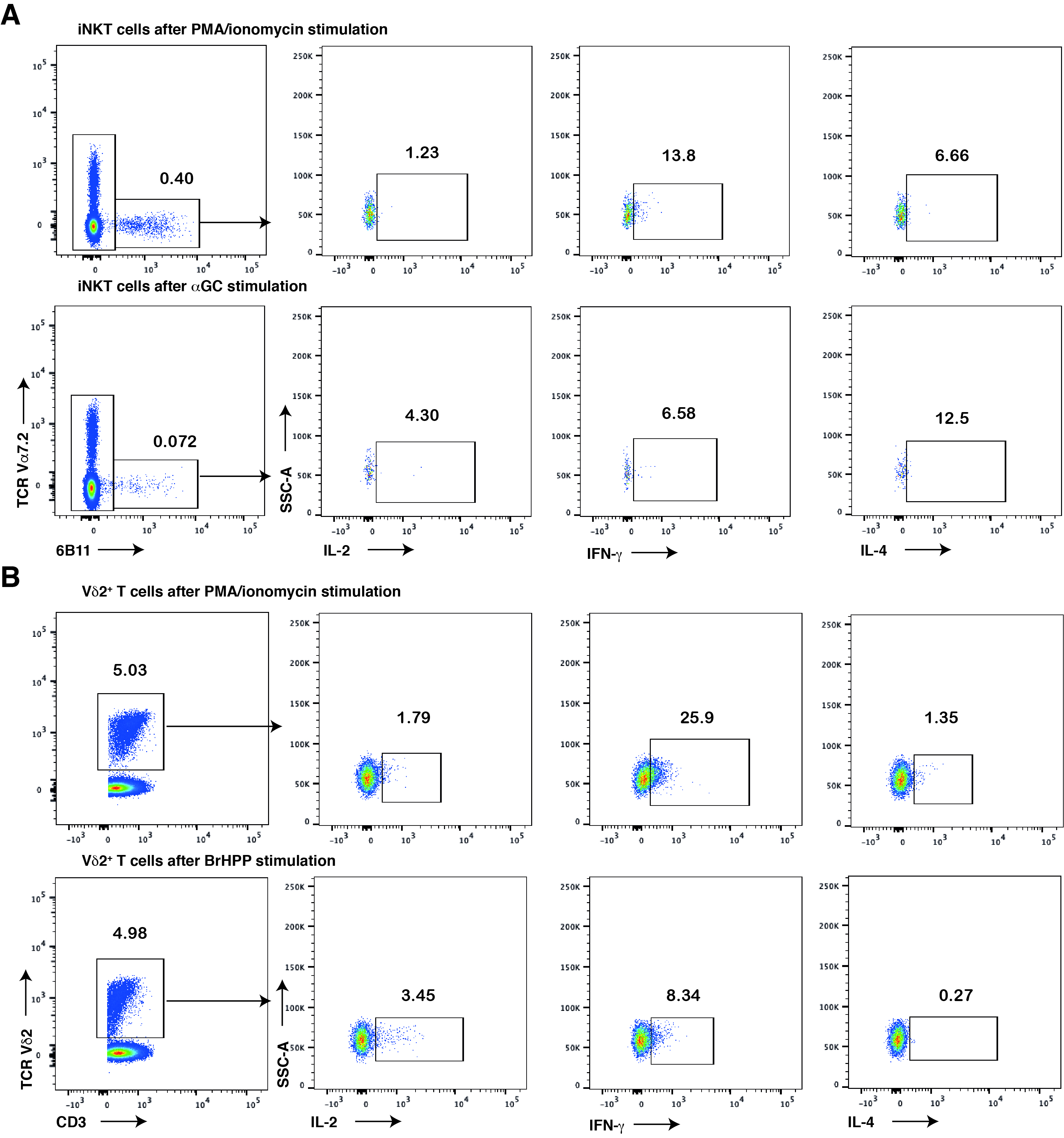

Supplement: Supplementary Figure 4 — Representative flow cytometry plots of activated iNKT and Vδ2+ T cells. (A) iNKT cells, identified as live CD19−CD3+TCRVα24-Jα18+ cells, were activated with either PMA/ionomycin (upper row) or α-GC (lower row) and cytokine production quantified by intracellular cytokine staining. (B) Vδ2+ T cells, identified as live CD19−CD3+TCRVδ2+ cells, were activated with either PMA/ionomycin (upper row) or BrHPP (lower row) and cytokine production quantified by intracellular cytokine staining. Numbers indicate % of gated subsets. [file Image_4.JPEG]
